# Mimicking Embedded Vasculature Structure for 3D Cancer on a Chip Approaches through Micromilling

**DOI:** 10.1038/s41598-017-16458-3

**Published:** 2017-12-01

**Authors:** L. Wan, J. Skoko, J. Yu, O. B. Ozdoganlar, P. R. LeDuc, C. A. Neumann

**Affiliations:** 1 Department of Mechanical Engineering, Carnegie Mellon University, Pittsburgh, 15213 United States; 2 Department of Biomedical Engineering, Carnegie Mellon University, Pittsburgh, 15213 United States; 3 Department of Materials Science and Engineering, Carnegie Mellon University, Pittsburgh, 15213 United States; 4 Department of Pharmacology and Chemical Biology, University of Pittsburgh Cancer Institute, Magee Womens Research Institute, Pittsburgh, 15261 United States

## Abstract

The ability for cells to sense and respond to microenvironmental signals is influenced by their three dimensional (3D) surroundings, which includes the extracellular matrix (ECM). In the 3D environment, vascular structures supply cells with nutrients and oxygen thus affecting cell responses such as motility. Interpretation of cell motility studies though is often restricted by the applied approaches such as 2D conventional soft lithography methods that have rectangular channel cross-sectional morphology. To better simulate cell responses to vascular supply in 3D, we developed a cell on a chip system with microfluidic channels with curved cross-sections embedded within a 3D collagen matrix that emulates anatomical vasculature more closely than inorganic polymers, thus to mimic a more physiologically relevant 3D cellular environment. To accomplish this, we constructed perfusable microfluidic channels by embedding sacrificial circular gelatin vascular templates in collagen, which were removed through temperature control. Motile breast cancer cells were pre-seeded into the collagen matrix and when presented with a controlled chemical stimulation from the artificial vasculature, they migrated towards the vasculature structure. We believe this innovative vascular 3D ECM system can be used to provide novel insights into cellular dynamics during multidirectional chemokineses and chemotaxis that exist in cancer and other diseases.

## Introduction

The ability to develop 3D systems that mimic physiologically relevant conditions will provide improved experimental environments to study complex cell and tissue response. Anatomical simulation of the chemical microenvironment includes nutrients and oxygen, which are delivered to cell tissues through a net of vasculature that create important biomolecular gradients within the tissue. As these structures lead to differences in concentration of chemical stimuli, individual cell responses vary in biological processes such as cell growth, migration and differentiation^1^. While these affect a diversity of diseases, recent cancer research has shown that gradients of proteins and oxygen generated through natural diffusion play an essential role in angiogenesis and metastasis by stimulating cancer cell chemotaxis^1–3^. *In vitro* biomolecular gradients of growth factors such as epidermal growth factor (EGF) in 3D hydrogels also presents chemical concentrations with different spatial and temporal distributions that affect cancer cell response^4^. To generate biomolecular gradients *in vitro*, methods including biological hydrogels^5^, micropipettes^6^, transwell assays^7^ and microfluidics^8–11^ have been developed with unique functions. Biological hydrogels provide an *in vivo* environment for free diffusion of chemicals in 3D matrices with spatial control provided by the biomolecule source. However, microfluidic systems offer an important advantage of mimicking complex geometries such as vasculature like structures, thereby facilitating more definitive quantification, and importantly, reproducibility^12,13^.

Microfluidics have been used in a variety of biological studies, yet one area, which is particularly useful in tissue biomimetics, is in creating vasculature like structures. Microfluidic channels have limitations such as often being limited to 2D soft lithography approaches^14^. Despite the limitation of 2D soft lithography, groups have attempted to create three-dimensional designs of channels with the soft lithography method^15^. Multiple-step micro-molding used to produce channel cross-sections to fabricate complex structures through approaches such as layer by layer fabrication are still limited in resolution though. Multilayer fabrication also presents challenges in providing smooth 3D channels due to stacks producing several uneven edges^16^. Another approach that has been explored recently is 3D printing to fabricate micro channels. While considerable advances have been made, this technique is often limited by relatively low resolution for 3D biosystems and challenges with curved or circular features due to the resolution with printing approaches^17,18^. 3D bioprinting recently produced endothelial and mesenchymal stem cell embedded tissue, with circular channels, yet the 3D channels designed were limited by the rigidity and stiffness of the biomaterials, and were 500 μm in diameter^19^. Thus our goal is to use microfluidic inspired approaches but implement micromachining and micromolding to address these issues and create vascular structures in 3D ECM matrices for examining cancer cell migration.

Cell migration is an important feature of cancer progression and metastasis. Unfortunately, examining cell motility *in vitro* has been challenging with many studies limited to using rigid two-dimensional substrates that have limited reflection of physiological relevant conditions^20–22^. Significant differences between 2D and 3D cell response are known^23–25^. For example, tumor progression and migration is related closely to the 3D structure of the ECM, which underscores the importance for 3D structures that mimic physiological architecture as closely as possible^26^. In addition, most systems to date lack an ability to control biomolecular gradients in 3D gels with respect to breast cancer cell motility and chemotaxis^27^, as well as spatial and temporal influences provided by a 3D vascular embedded system. *In vivo*, tumor growth often depends on a 3D vascular system that supplies rapidly growing cells nutrients and oxygen. This 3D challenge is underscored as rapid cell division is a hallmark of many cancers, and the need for oxygen from the 3D vasculature is high and cannot be met appropriately in a timely fashion. Therefore, cancer cells develop mechanisms allowing them to grow in hypoxic conditions which changes their behavior to a more aggressive phenotype from a cell motility perspective that stimulates angiogenesis by producing vascular endothelial growth factor (VEGF)^28,29^. The formation of 3D biomolecular gradients can evolve because of the delay in new vasculature development, which contributes to tumor heterogeneity.

In this paper, we developed a micromachining method for fabricating double parallel circular channels embedded within a 3D collagen matrix. When breast cancer cells were embedded inside the scaffold, stimulation profiles were controlled through diffusion of fetal bovine serum (FBS) between the channels to generate gradients that enhanced cell migration. Our device can directly be applied to examine cell viability, proliferation and motility, in response to chemical stimuli in a controlled 3D environment. Compared with most other microfluidic designs with PDMS channels^30,31^, this novel device mimics physiologically relevant 3D environments that allow investigation of cancer cells behavior during multidirectional chemokineses and chemotaxis. Thus, future applications may also include drug screening methods.

## Results

### 3D extracellular matrix cancer gradient microfluidic design

**Figure 1:**
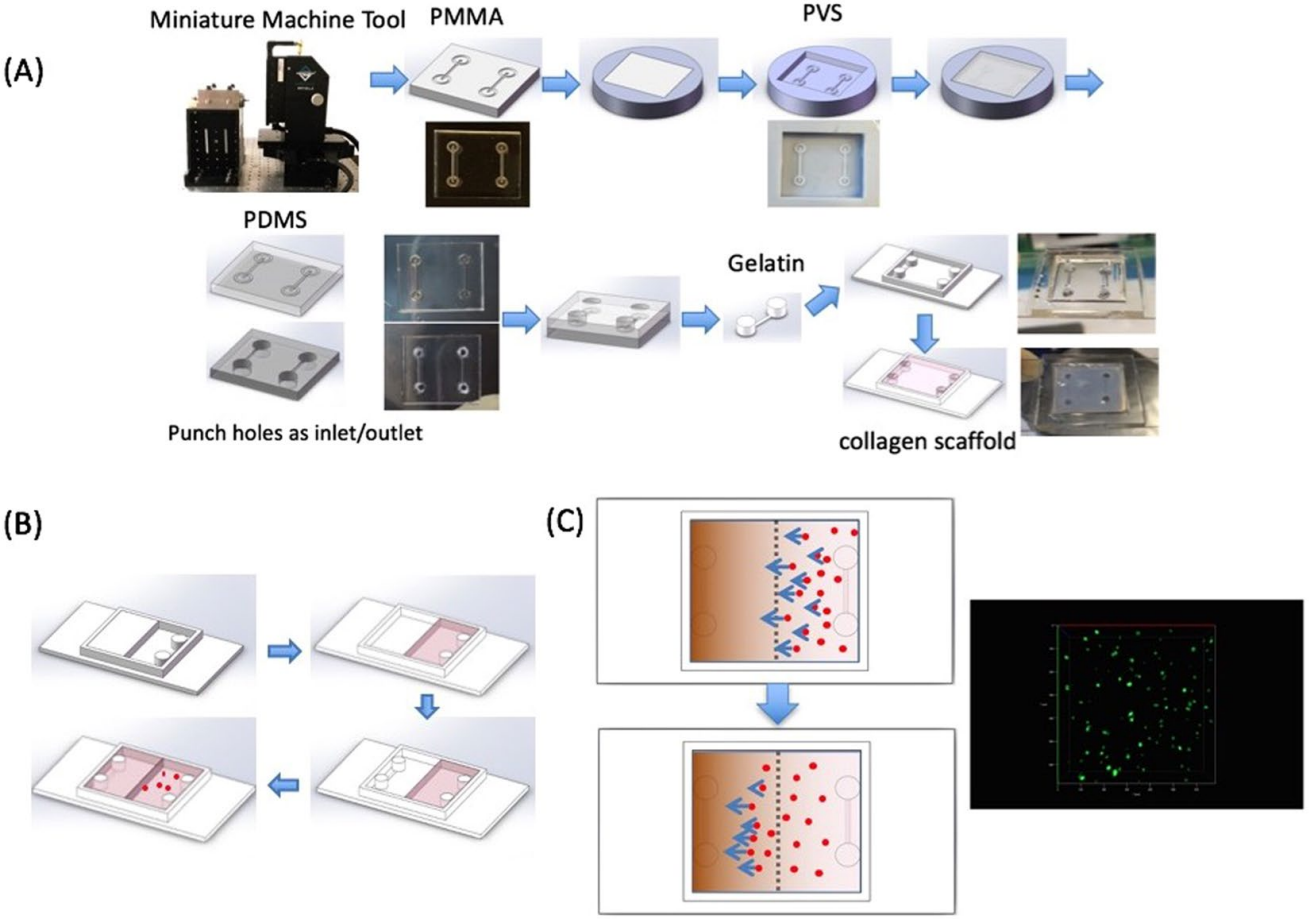
Mimicking embedded vasculature structure for 3D cancer on a chip approaches through micromilling. Fabrication and characterization of microfluidic device. (**A**) Schematic overview of collagen device fabrication with microfluidic channels for cancer cell motility. Semicircular channels are micromilled on PMMA master mold. Positive patterns are molded on PVS, and negative pattern are regained through PDMS. A sacrificial gelatin template is obtained by injecting liquid gelatin into the PDMS mold gelled. With the gelatin template located in the chamber, collagen is added and incubated for collagen crosslinking and gelatin melting, leaving microfluidic channels embedded in the ECM. (**B**) Two stage fabrication approach to distribute cells uniformly in one half of the ECM. Collagen without cells is filled in half of the chamber, followed by filling collagen with cancer cells into the other half. (**C**) Experimental design and confocal imaging with MDA-MB-231 cancer cells initially located in the right half of system. The cells migrate to the left due to the FBS gradient.

We developed a simple and reproducible method to fabricate 3D ECM into a chip with circular channels embedded in the scaffold for creating gradients for cancer cell responses. Collagen type I was selected as an ECM, as it is a natural component of many 3D matrices in various tissues^32^. Gelatin was fabricated to serve as a sacrificial layer due to its thermo-reversible property and biocompatibility. To create circular channels within the ECM, we fabricated cylindrical gelatin templates (500 μm in diameter) with inlet and outlet handles through a series of processes with our micromachining approach (Fig. 1). A PMMA mastermold was first machined to create semi-circular channels with a ball-shape drill bit through micromachining. Polyvinyl siloxane (PVS) was applied to create a positive mold (Fig. S1) and a PDMS negative mold was produced subsequently through a reverse molding process. Liquid gelatin was injected into the PDMS mold at 37 °C and cooled to gel (Fig. S2). Gelatin templates were then placed into the chip chamber and covered with a mixture of collagen and cells (Figs 1B, S3). As the collagen polymerized at 37 °C, the gelatin template melted and circular channels remained. The design of double parallel channels in the ECM allowed for spatiotemporal control in creating a gradient. The collagen concentration of the 3D ECM was 5 mg/ml to maintain the structural stability of the microfluidic channel. Although this resulted in a slighter higher ECM stiffness as found *in vivo*, it is still within the range of physiological relevance^33,34^.

To test if we could develop a biomolecular gradient in the microfluidic chip chamber, chemotaxis-induced cell migration of the breast cancer cell line MDA-MB-231 through the collagen was examined (Fig. 1C). To accomplish this, the chamber was divided in two halves. One chamber half was filled with collagen and allowed to solidify before the other half was filled with a collagen-cell suspension. Cell migration was observed after DMEM medium with various FBS concentrations were introduced into both channels to develop a biomolecular FBS gradient.

### Chemical gradient characterization with rhodamine 6G

**Figure 2:**
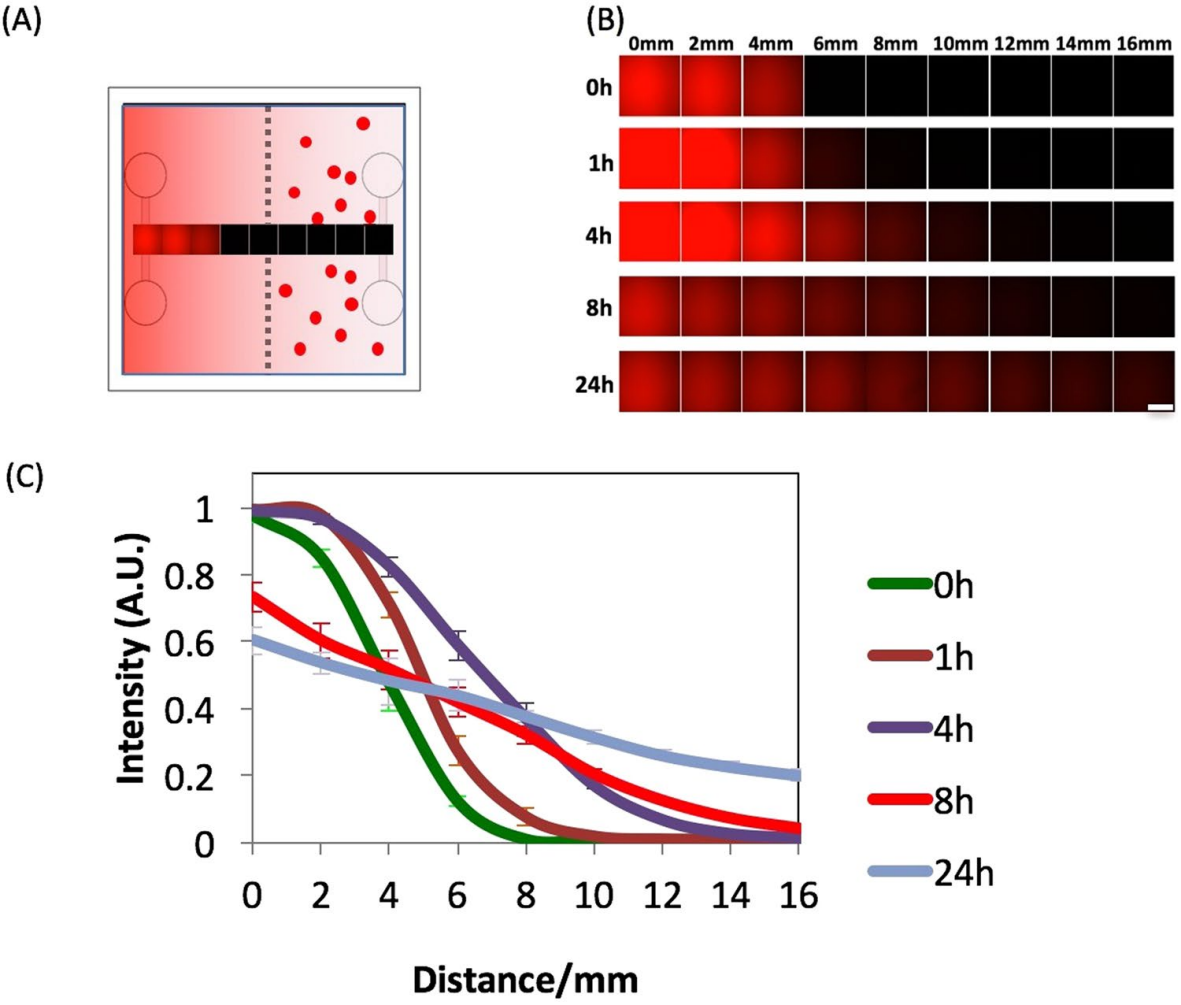
Analysing diffusion for creating chemical gradients in our approach using Rhodamine 6G. (**A**) Schematic of our 3D ECM microfluidic approach with the left channel filled with rhodamine 6G at t = 0 hr. Images are captured at 2 mm spacing from each other. (**B**) Images showing the intensity distribution profile of rhodamine 6G changing over 24 hr by diffusion captured at 2 mm spacing. Scale bars are 300 µm. (**C**) Normalized intensity profile of rhodamine 6G fluorescence intensity after 0, 1, 4, 8, or 24 hr.

Rhodamine 6G was utilized as an indicator for diffusion due to its fluorescent properties and comparable molecular size (471 g/mol) with components of FBS, such as epidermal growth factor (EGF)^35,36^. 30 μl of rhodamine 6G was injected into one channel (initiating channel) and allowed to diffuse towards the opposite end of the chamber for 24 hr (Fig. 2A). The intensity profile of rhodamine 6 G was quantified by confocal microscopy as an indicator of local concentration (Fig. 2B,C). Rhodamine 6G was observed to diffuse significantly towards the opposite channel within 4 hr, forming an approximate linear profile after 8 hr. Without adding any additional rhodamine 6G, its concentration at the initiating channel slowly dropped to 60%, while the concentration at the opposite channel increased by 20% after 24 hr (Fig. 2C). Similarly, we applied fluorescently labeled EGF as an indicator for testing the diffusion (Fig. S4).

Previously, Naumen *et al*.^35^ used a method to estimate diffusion coefficient of multiple growth factors such as Bovine serum albumin (BSA) and EGF, which were similar to our diffusion coefficients. For example, BSA has a diffusion coefficient of 0.72 × 10^−6^ cm^2^/s in an aqueous environment, while EGF has diffusion coefficient of 1.34 × 10^−6^ cm^2^/s and rhodamine 6 G has 4.14×10^−6^ cm^2^/s^34,35^. As these diffusion coefficients are comparable, the growth factors provided through medium in the channels would develop a similar gradient profile.

### Cell viability within the collagen system under gradients

**Figure 3:**
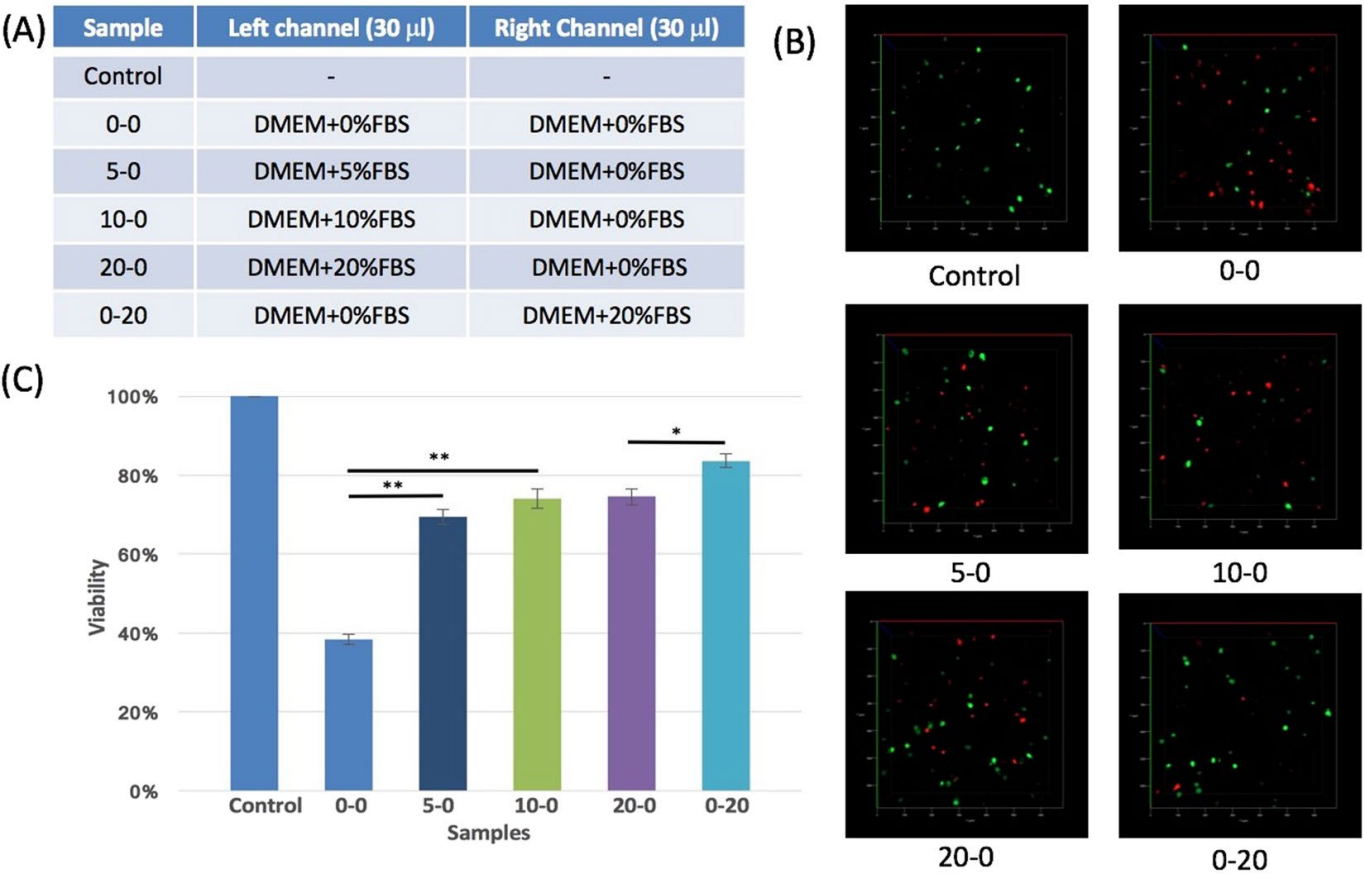
Viability of MDA-MB-231 cells within the 3D collagen systems. (**A**) Controlled FBS concentrations were placed in the left and the right channels independently including 0, 5, 10, or 20% FBS indicated on the table. (**B**) Fluorescent images of live and dead cells in each sample. Dead cells are stained red with EthD-1 and all green cells are indicated with GFP. (**C**) Normalized viability of cells in each sample with trypan blue stain test.

To examine compatibility of the device, MDA-MB-231 cells were cultured in the ECM and tested for viability over 24 hr. We compared different FBS chemotaxis gradient profiles of five samples with varying FBS concentrations to a control sample with polymerized collagen (Fig. 3). The control sample was tested immediately after collagen polymerization. For the other five samples, we injected 30 μl of DMEM with different FBS concentrations into both channels, and incubated them at 37 °C for 24 hr. For example, sample 0–20 has DMEM without FBS injected in the non-cell side channel (thus “0” in the 0–20 sample) and DMEM with 20% FBS injected to the uniform-cell side channel (thus “20” in the 0–20 sample). To determine the average viability of cells in the device, the collagen scaffolding incubated after 24 hr was dissolved by Type I collagenase and stained with trypan blue for live cell counting. Collagenase specifically degrades the 3D collagen matrix without harming cell membranes^37^. Cell viability was reduced with decreasing concentrations of FBS due to the necessity of various components within the serum to support cell survival based on trypan blue stain test (Fig. 3C). In sample 0–0, only 40% of cells remained alive within 24 hr without any FBS supply. With 3 μl of FBS (10% of 30 μl DMEM) in sample 5–0, the viability increased to over 70% in 24 hr. Higher FBS concentration increased viability of cells in general, up to 84% in sample 0–20.

As a second approach, staining of the ECM with ethidium homodimer-1 (EthD-1) allowed the detection of dead cell distribution directly through confocal microscopy (Fig. 3B). All cells were fluorescently imaged as green due to EGFP, while dead cells were stained and emitted a red fluorescent signal due to EthD-1. The viability from the EthD-1 studies supported the trypan blue findings (Fig. S5). Cell viability depended greatly on ECM thickness as a thicker ECM reduced cell viability (Fig. S6). 2 mm thickness of ECM was chosen for migration testing as it provided good cell viability, while the stability of the channel (500 μm diameter) was not compromised.

### MDA-MB-231 motility with FBS gradient

**Figure 4:**
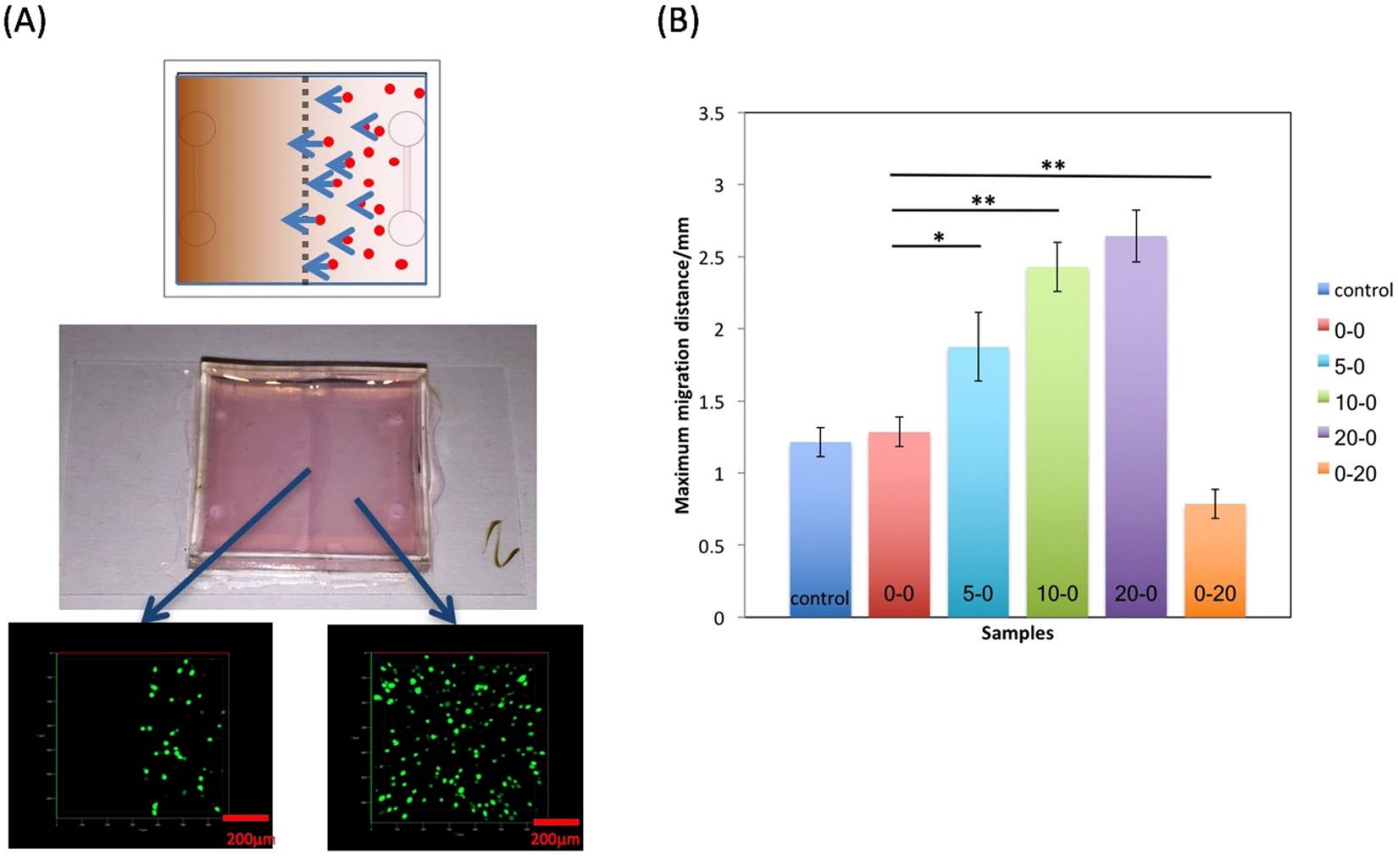
Migration of MDA-MB-231 cancer cells within 3D ECM microfluidic gradient system. Distance detected in all samples. (**A**) Representative device with cells uniformly distributed in the right half of chamber at the initial time point. The cells move due to the FBS gradient in the 3D collagen system toward the left side where no cells are present over 24 hours. (**B**) Maximum migration distance of the cancer cells for each controlled FBS gradient concentration. Controls show similar movement to 0–0 sample with little movement diffusion distance of cells. Significant migration is observed in samples with positive FBS gradients (5–0, 10–0 and 20–0). Reverse migration is observed with negative FBS gradient (0–20).

Cell migration is essential for a diversity of biological processes^38^, and in this work we focused on cancer cell motility. Tracking cell migration in 3D with controlled vascular structures is challenging. Through our approach, a clear vertical collagen boundary visible at the center of our collagen scaffolding was created through a two-step collagen filling processes (Fig. 4). This two-step process allowed uniform cells spreading and reliable measurement of cell migration distance across the boundary using confocal microscope imaging (Figs S7–S10). Cell motion was uniform towards higher FBS gradients when observed along the length of the channels, as the FBS gradient was approximately parallel between the channels. Since cell migration towards FBS appeared to form a homogenous front (Fig. 4A), we defined the distance between the migration front and the middle of device, where the two halves of the collagen meet, as the maximum distance traveled for all samples. For 5–0, 10–0 and 20–0 samples, FBS gradients (with the higher gradient on the left side of the system) developed across two parallel channels, leading to significant cell migration in 24 hr. Migration distance was around 1.25 mm for sample 10–0 and 20–0. or an average of 0.87 μm/min during that time (Fig. 4B). For the 0–20 sample, we generated a reverse gradient of FBS (with the higher gradient on the right side of the system) and observed the cell migration moving 0.7 mm in 24 hr compared to our control samples. Cells exhibited migration toward the higher FBS concentration.

## Discussion

We designed an ECM microenvironment simulator and applied our design to initially test cell migration in response to a biomolecular gradient in 3D. Cell migration was examined by testing cancer cell chemokineses and chemotaxis. This approach is of particular interest as modeling of cancer cell invasion *ex vivo* is still limited. The motility of cancer cells is directly affected by the ECM environment in which it resides^39^. Thus, the goal of this study was to create a microsystem that moves away from inorganic polymers such as PDMS, to create a more physiologically relevant microenvironment that enables the examination of cell behavior. Zerantonakis *et al*.^40^ reported detection of cell migration with chemotaxis, however, we fabricated microvasculature directly in ECM, instead of using PDMS microfluidic channels as the fluid flow platform. Collagen is more physiologically relevant than PDMS, which may help to mimic better *in vivo* condition where growth factors and other chemicals diffuse from blood/lymphatic vessel systems to tumors and involve the ECM. We also wanted to study how gradients affect cellular responses in ECM environments. For example, often chemotactic cellular phenotypes are tested using unidirectional approaches with inorganic polymers^41^. However, as gradients fluctuate constantly within a tumor microenvironment, this limits supplies for the cellular environment for cancer cells, which are constantly forced to adapt in order to survive. These mechanisms promote tumor heterogeneity and aggressiveness. Therefore, our novel system will be extremely useful as it: a) provides a physiological ECM in which inorganic polymers were replaced by collagen, and b) channels were embedded that allow convenient controlled generation of the desired chemoattractant gradient. This way, embedded cells can be examined for potentially previously unobserved cell behaviors by mimicking tumor-microenvironment related *in vivo* conditions.

Cell response in collagen matrix is dependent on a number of factors including matrix thickness and stiffness. These factors affect many components including the nutrient and oxygen supply in the collagen matrix, which is not only limited by its low diffusion speed from the air, but also by cellular consumption at the surface that decreases the oxygen available to cells within the matrix^32^. This can cause challenges with 3D experiments. We found that increasing the matrix thickness can reduce cell viability significantly (Fig. S6).

Migration in our experiments was specifically studied by pre-treating cells with mitomycin C prior to cell seeding. This enabled elimination of cell proliferation within the ECM matrix being mistaken as cell migration. Our migration findings showed cells responded depending on the relative FBS gradients to which they were exposed. Without the gradient, both the control and 0–0 sample showed 1.25 mm of cell movement (Fig. 4B). This could be due to collagen diffusion. Also, significant migration in the 20–0 sample was observed with a greater FBS gradient between the two channels. This response was likely due to the chemotaxis response of the cells to this gradient. Differences between 10–0 and 20–0 samples were not as large, which could be because the FBS gradient in 10–0 was high enough to trigger cell migration with maximum speed. With an opposite gradient, the 0–20 sample showed reverse cell migration for approximately 0.7 mm compared with control and 0–0 samples, indicating the ability for cells to sense the direction of FBS gradient and move in the opposite direction. In these experiments, MDA-MB-231 cells responded to various FBS gradients and migrated in our 3D ECM microfluidic approach toward higher concentrations.

## Conclusions

We developed an approach to create a 3D collagen matrix microfluidic system with multiple curved channels embedded within. Importantly, in comparison to other PDMS microchannel devices, this novel design contains embedded vasculature structures within a 3D collagen matrix, that have physiologically relevant polymers instead of the almost always inorganic polymers like PDMS. MDA-MB-231 cancer cell migration with respect to the FBS gradient across the channels was tested and showed that the cancer cells migrated toward the higher gradients when presented with different concentrations of FBS across the collagen through the dual microfluidic channel approach. The 3D geometric features similar to the vascular system were utilized to provide better mimicking of *in vivo* environments through the integration of cells within the ECM. Our approach could be further applied to observe cell migration in response to hypoxia gradients and other environmental regulated conditions. We also believe our approach will contribute in the future to correlating *in vitro* experiments with *in vivo* responses for high throughput *ex vivo* cancer treatments such as in organ on a chip diagnostic approaches and as testbeds for drug screening.

## Methods

### Microfluidic chip chamber and gelatin template fabrication

The chip chamber was fabricated with a 2.5 mm PMMA board (TAP plastic) that was cut into rectangular boundaries with a laser cutting system (Epilog, CO), and then adhered to a coverslip with Optical Adhesive (Norland products Inc., NJ), (Fig. 1). The Chamber was designed with dimensions of 17 mm × 19 mm × 2 mm, so the overall volume was 646 μl. Gelatin sacrificial templates were fabricated as previously described^42^. The PMMA mastermold was fabricated through micromachining with a high precision machine tool (MMT) to generate parallel semicircular channels (Fig. S1). The PMMA master was then molded to produce positive replicas with Polyvinyl siloxane (PVS, R-2364, Silpak Inc., Pomona, CA), which was subsequently reverse molded with PDMS to regain negative channel structures. Next, one PDMS mold was punched at both ends of the channels to generate inlets and outlets. PDMS molds were then aligned and attached face-to-face to complete a circular channel. 12% gelatin (Sigma) solution was then pre-incubated at 37 °C for dissolving and injected into the channel. After the gelatin was solidified at 4 °C for 1 hr, it was removed from the PDMS mold and stored in 1× PBS at 4 °C.

### Cell culture and device fabrication

For the breast cancer cells, MDA-MB-231 cells (ATCC) were transduced with viral particles produced in HEK 293 T cells (ATCC) as described^43^. 1 µg of pLVX-EGFP-IRES-Hyg, psPAX2 and pMD2.G (3:2:1 DNA molar ratio) were transfected with Fugene 6 (Promega) (6:1 Fugene:DNA ratio) into 1 × 10^5^ 293 T cells in a 6-well plate in 2 ml OptiMEM media (Gibco) for 16 hr at 37 °C with 5% CO_2_. Following transfection complex removal, 2 ml DMEM (Gibco) supplemented with 10% FBS (HyClone), 100 units/ml penicillin (Gibco), 100 mg/ml streptomycin (Gibco), non-essential amino acids (Gibco) and 2 mM l-glutamine (Gibco) was added to the 293 T cells to produce viral particles for 48 hr. MDA-MB-231 cell transduction was performed in culture media containing 8 µg/ml polybrene (Sigma) for 24 hr at 37 °C with 5% CO_2_. After 24 hr recovery, MDA-MB-231 enhanced green fluorescent protein (EGFP) positive cells were selected with 500 µg/ml hygromycin (Gibco) in culture media for 1 week.

Cell migration devices were fabricated in two stages. First, half of the collagen vascular matrix without the cells was first developed by blocking the other half of the collagen with a PMMA piece. The gelatin template was first located at edge of the half chamber and then 23 μl of 1 N NaOH, 250 μl of 10×PBS (ThermoFisher Scientific), 971 μl of DMEM and 1250 μl of 10 mg/ml collagen (High concentration collagen type I, Corning) were mixed uniformly at 4 °C, in order to obtain a final collagen concentration of 5 mg/ml. Next 323 μl (50% of the overall chamber volume) mixed solution was added to fill each chamber to completely cover the gelatin template. The device was incubated at room temperature for 1 hr followed by 37 °C for 30 min for collagen polymerization.

The second half of the chamber was designed with a uniform cell distribution within the collagen matrix and fabricated by removing the PMMA blockers. MDA-MB-231 cells were pretreated with mitomycin C (Sigma) for 2 hr to block proliferation. The cells were then dissolved in 971 μl of DMEM and stirred with 23 μl of 1 N NaOH, 250 μl of 10×PBS and 1250  μl of 10 mg/ml collagen, with a final cell density of 1.7 × 10^5^ cells/ml and 5 mg/ml collagen concentration. Another gelatin template was placed into the chamber at the edge and 323 μl of the mixed solution was added to the half chamber. The device was incubated at room temperature for 1 hr and moved to 37 °C for 30 min for collagen crosslinking and gelatin dissolution. Liquid gelatin could be removed with a P200 pipette, leaving channels within the collagen (Figs S2, S3).

### Rhodamine 6G and EGF diffusion test

30 μl of rhodamine 6 G (Sigma) at a concentration of 10^−4^ g/ml^44^ was added into one channel of each device. Devices were incubated at 37 °C for 0, 1, 4, 8 or 24 hr. The devices were then immediately imaged with a confocal microscope (Axio Observer Z1 Microscope System, Zeiss). The images were then processed linearly between the channels to measure average intensity and thus obtain an intensity profile. Similarly, 30μl of fluorescently labeled EGF (Biotinylated, complexed to Alexa Fluor™ 647 Streptavidin, ThermoFisher Scientific) at a concentration of 10μg/ml was added to one channel and examined at 0, 1, 4, 8, 24 hrs of diffusion (Fig. S4).

### Sample design and viability test

To generate different FBS gradient profiles, we analyzed the viability and cell migration in our systems. The control sample was immediately tested after the collagen matrix was crosslinked. All other samples had both channels injected with 30 μl of DMEM supplemented with different FBS concentrations and after 24 hr, the viability of cells was examined in the microfluidic device by using Trypan blue and Ethidium homodimer testing (see below).

### Trypan blue stain test

The entire collagen slabs were separated from the chamber and cut into smaller pieces. These pieces were then added into a 15-ml conical tube with 5 ml of a washing solution (1xPBS supplemented with 5% FBS) and centrifuged at 1500 rpm for 5 min. Supernatants were removed and 1 ml of Type I collagenase (ThermoFisher Scientific) solution (10 ml DMEM with 5% FBS and 30 mg collagenase) was added into the tube. After 1 hr incubation at 37 °C, collagen pieces were completely dissolved. After another centrifugation step, supernatants were removed, leaving 500 μl of solution in the tube. Then 500 μl of 0.4% Trypan blue (ThermoFisher Scientific) was added and incubated at room temperature for 5 min. Using a hemocytometer, stained (dead) and unstained (live) cells were counted for each sample.

### Ethidium homodimer-1 (EthD-1) stain test

200 μl of EthD-1 (ThermoFisher Scientific) was added on top of each chamber and incubated at room temperature for 1 hr to allow diffusion through the collagen. Then each device was rinsed twice with 200 μl of 1× PBS for 30 min. Images were immediately taken with the confocal microscope. Green fluorescent cells (EGFP positive) were counted as live cells and red cells (red fluorescence by EthD-1) were considered dead. Cell viability of each sample was calculated based on these results.

### Migration assay

Control sample was fixed with 200 μl of 4% Paraformaldehyde (PFA) solution immediately after fabrication. Five other samples were injected with corresponding DMEM with FBS solutions and incubated at 37 °C for 24 hr. All samples were then immediately imaged using the confocal microscope. Images were taken from the middle boundary and imaged towards the non-cell side to observe the maximum distance of migrated cells, with local cell migration spanning a distance of at least 0.5 mm. Migration distances were recorded for each sample as a quantitative indicator of cell migration rate.

## Electronic supplementary material

Supplementary Figures
