# Author Correction: Mimicking Embedded Vasculature Structure for 3D Cancer on a Chip Approaches through Micromilling

**DOI:** 10.1038/s41598-018-37927-3

**Published:** 2019-03-28

**Authors:** L. Wan, J. Skoko, J. Yu, O. B. Ozdoganlar, P. R. LeDuc, C. A. Neumann

**Affiliations:** 1 Department of Mechanical Engineering, Carnegie Mellon University, Pittsburgh, 15213 United States; 2 Department of Biomedical Engineering, Carnegie Mellon University, Pittsburgh, 15213 United States; 3 Department of Materials Science and Engineering, Carnegie Mellon University, Pittsburgh, 15213 United States; 4 Department of Pharmacology and Chemical Biology, University of Pittsburgh Cancer Institute, Magee Womens Research Institute, Pittsburgh, 15261 United States

Correction to: *Scientific Reports* 10.1038/s41598-017-16458-3, published online 1 December 2017

O. Burak Ozdoganlar was omitted from the author list in the original version of this Article. This has been corrected in the PDF and HTML versions of the Article, and in the accompanying Supplementary Figures file.

The Author Contributions section now reads:

L. Wan, P. R. LeDuc and C. Neumann conceived and designed the project. L. Wan conducted the experiments and analyzed the results. J. Skoko prepared the cells. O. B. Ozdoganlar and J. Yu prepared the acrylic mastermold. All authors reviewed/edited the manuscript.

