# Supplementary material for: Mimicking Embedded Vasculature Structure for 3D Cancer on a Chip Approaches through Micromilling
Source: Sci Rep. 2017 Dec 1;7:16724. doi: 10.1038/s41598-017-16458-3 (PMC5711800; doi:10.1038/s41598-017-16458-3)
Supplement: Supplementary file 1 — Supplementary Figures [file 41598_2017_16458_MOESM1_ESM.pptx]

## Slide 1
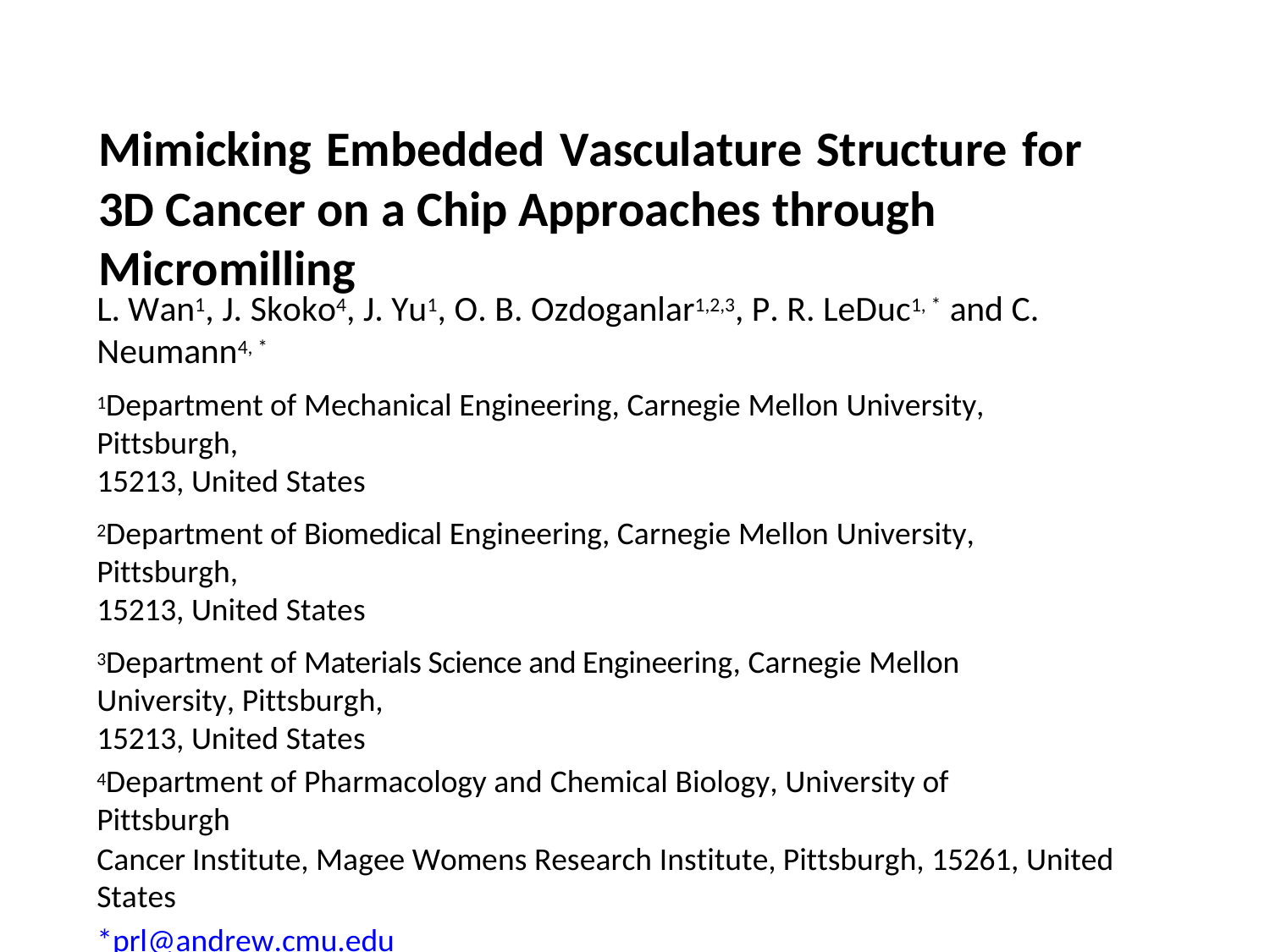

Mimicking Embedded Vasculature Structure for 3D Cancer on a Chip Approaches through Micromilling
L. Wan1, J. Skoko4, J. Yu1, O. B. Ozdoganlar1,2,3, P. R. LeDuc1, * and C. Neumann4, *
1Department of Mechanical Engineering, Carnegie Mellon University, Pittsburgh,
15213, United States
2Department of Biomedical Engineering, Carnegie Mellon University, Pittsburgh,
15213, United States
3Department of Materials Science and Engineering, Carnegie Mellon University, Pittsburgh,
15213, United States
4Department of Pharmacology and Chemical Biology, University of Pittsburgh
Cancer Institute, Magee Womens Research Institute, Pittsburgh, 15261, United States
*prl@andrew.cmu.edu
*neumannc@upmc.edu

## Slide 2
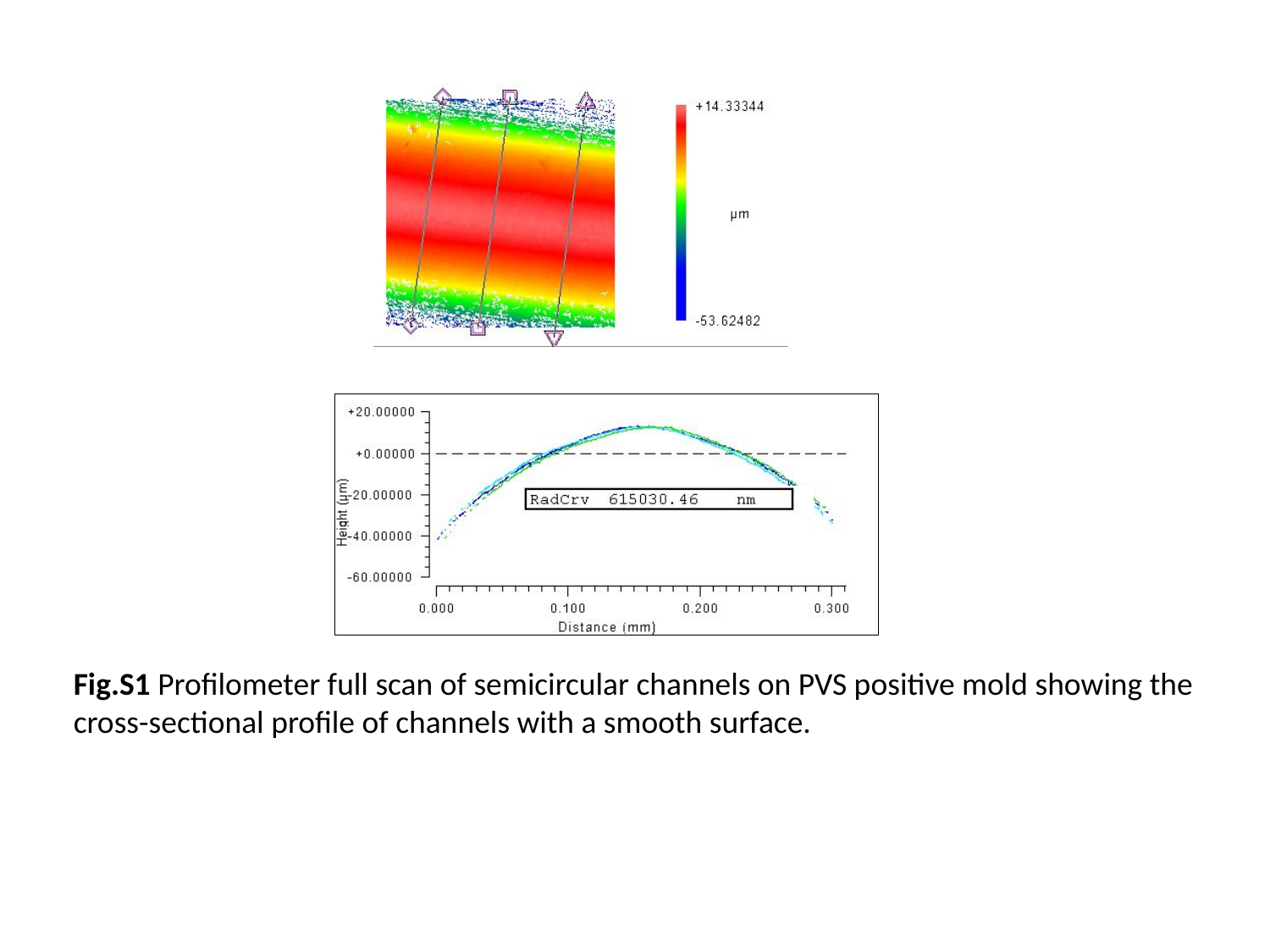

Fig.S1 Profilometer full scan of semicircular channels on PVS positive mold showing the cross-sectional profile of channels with a smooth surface.

## Slide 3
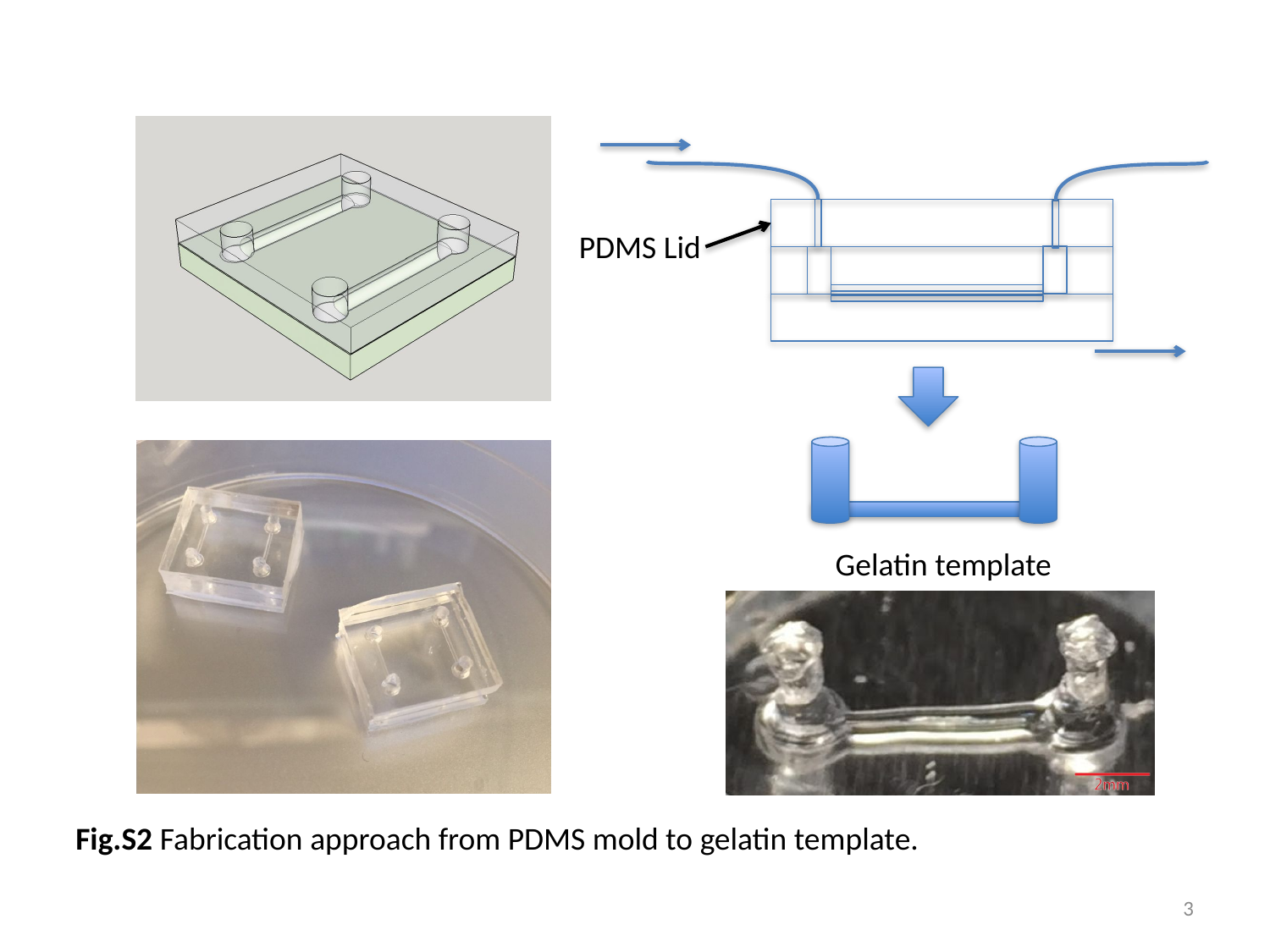

PDMS Lid
Gelatin template
Fig.S2 Fabrication approach from PDMS mold to gelatin template.
3

## Slide 4
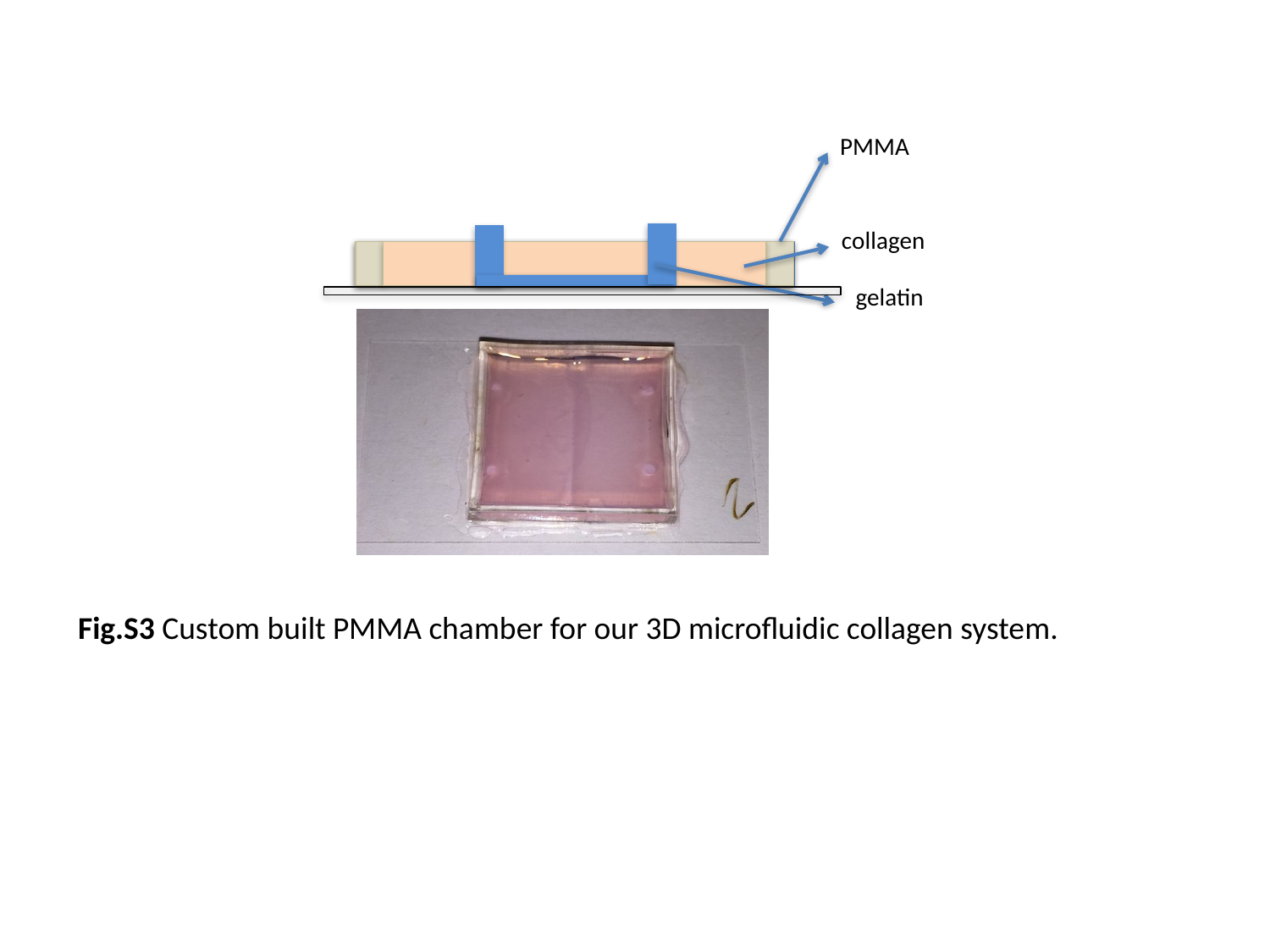

PMMA
collagen
gelatin
Fig.S3 Custom built PMMA chamber for our 3D microfluidic collagen system.

## Slide 5
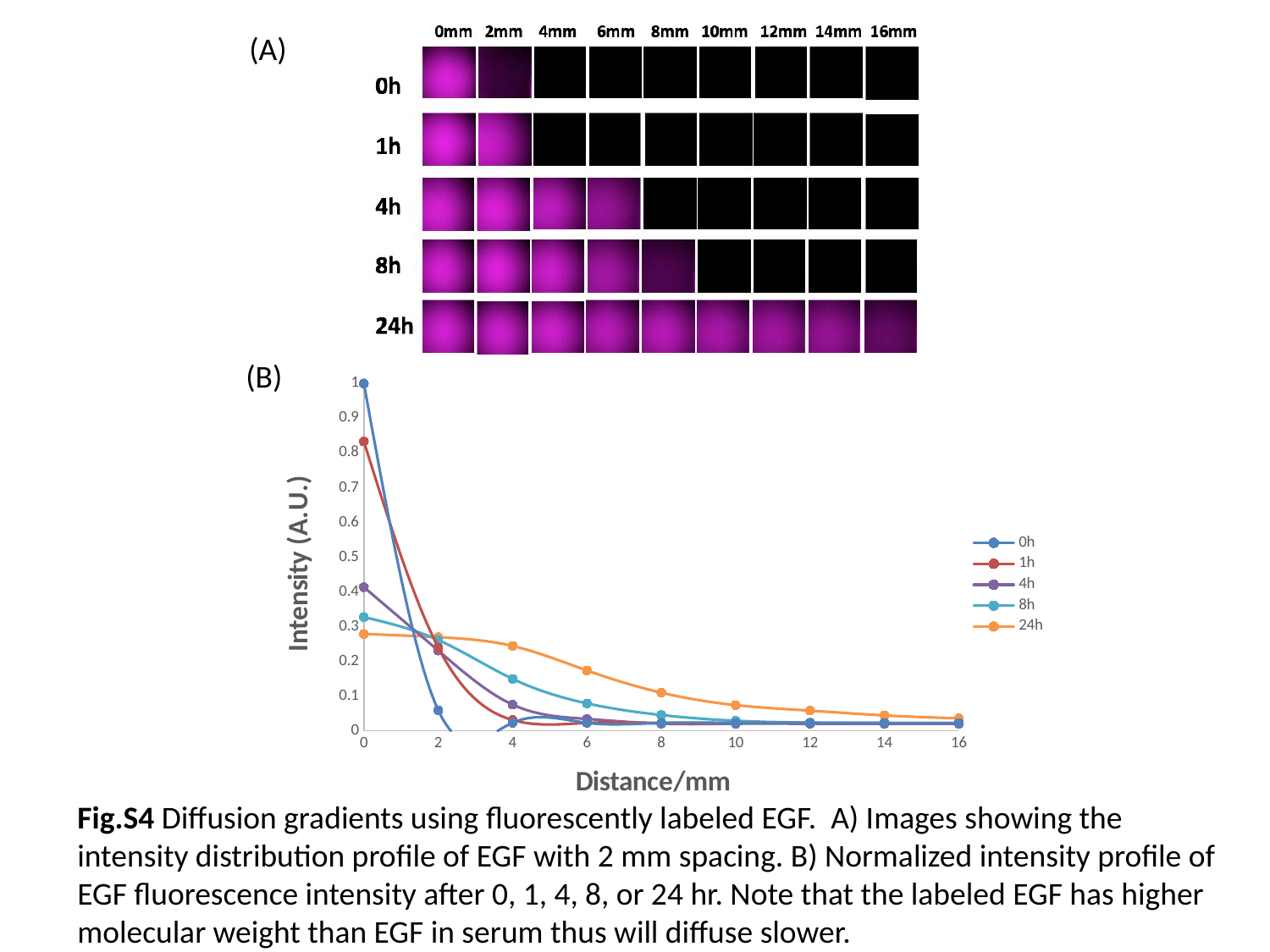

(A)
(B)
### Chart
| Category | 0h | 1h | | 4h | 8h | 24h |
|---|---|---|---|---|---|---|Fig.S4 Diffusion gradients using fluorescently labeled EGF. A) Images showing the intensity distribution profile of EGF with 2 mm spacing. B) Normalized intensity profile of EGF fluorescence intensity after 0, 1, 4, 8, or 24 hr. Note that the labeled EGF has higher molecular weight than EGF in serum thus will diffuse slower.

## Slide 6
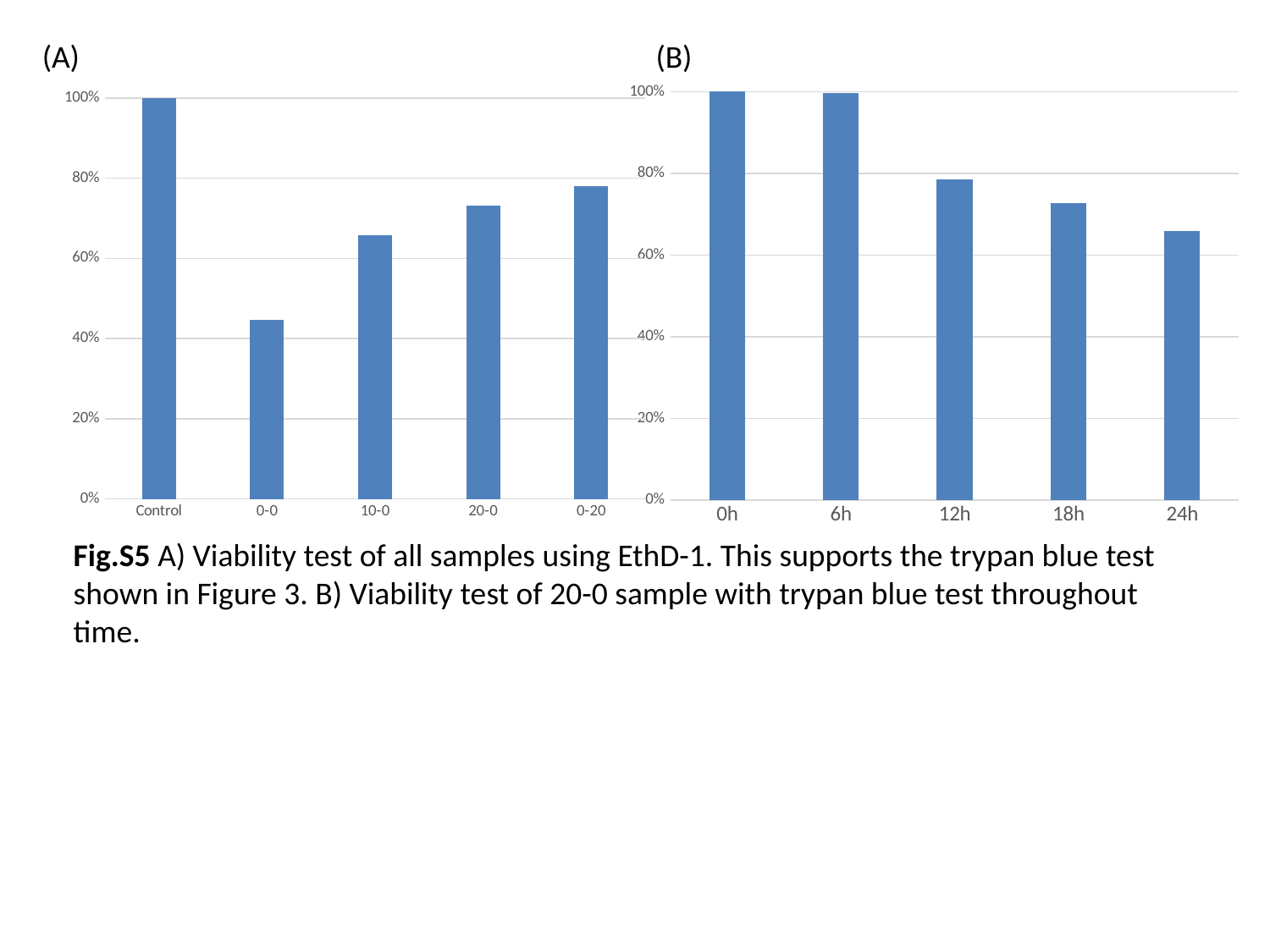

(A)
(B)
### Chart
| Category | |
|---|---|
| 0h | 1.0 |
| 6h | 0.997159090909091 |
| 12h | 0.785003317850033 |
| 18h | 0.728084415584416 |
| 24h | 0.658138268916712 |
### Chart
| Category | |
|---|---|
| Control | 1.0 |
| 0-0 | 0.446324951644101 |
| 10-0 | 0.658138268916712 |
| 20-0 | 0.731601731601732 |
| 0-20 | 0.779671717171717 |Fig.S5 A) Viability test of all samples using EthD-1. This supports the trypan blue test shown in Figure 3. B) Viability test of 20-0 sample with trypan blue test throughout time.

## Slide 7
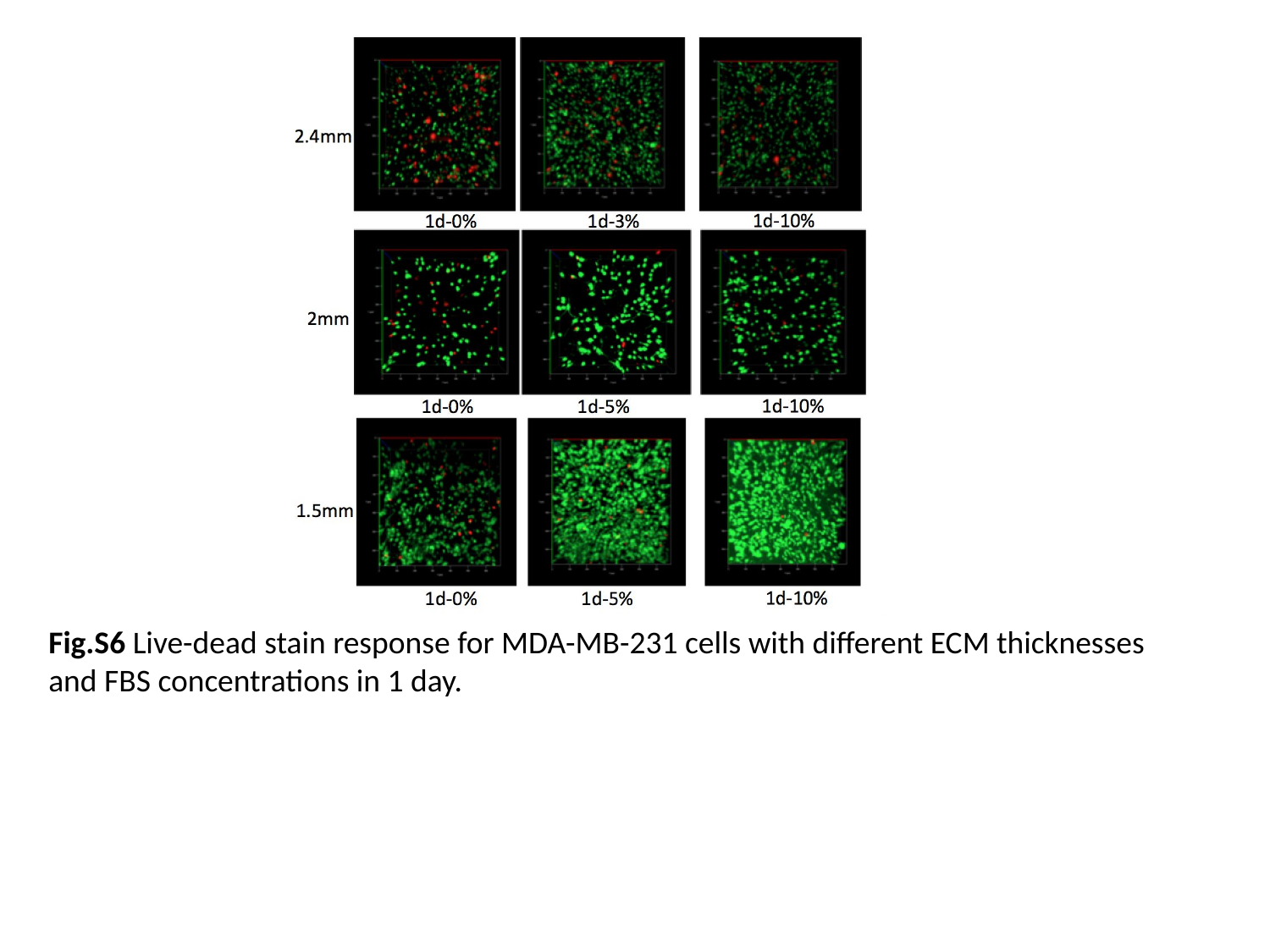

Fig.S6 Live-dead stain response for MDA-MB-231 cells with different ECM thicknesses and FBS concentrations in 1 day.

## Slide 8
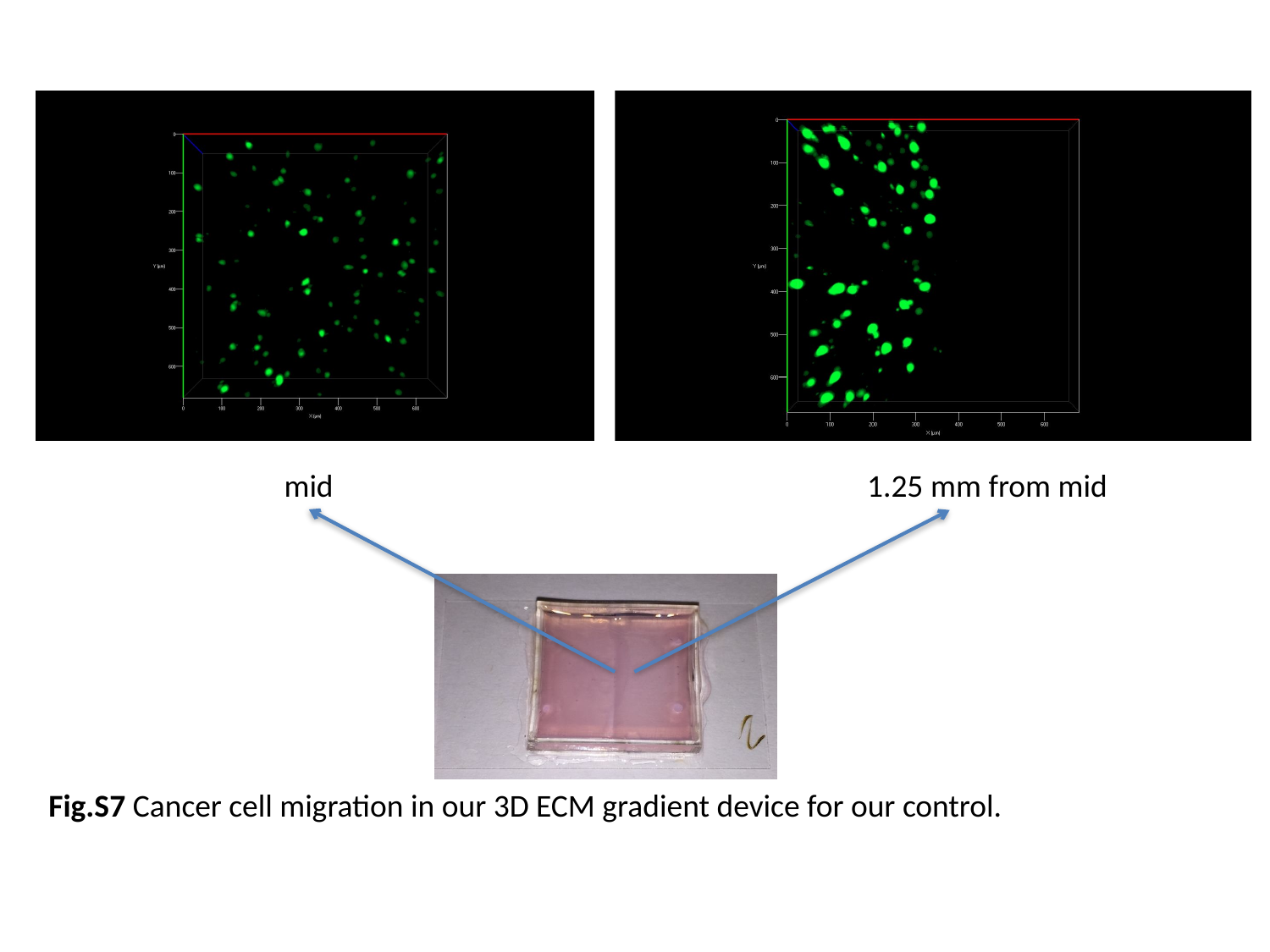

mid
1.25 mm from mid
Fig.S7 Cancer cell migration in our 3D ECM gradient device for our control.

## Slide 9
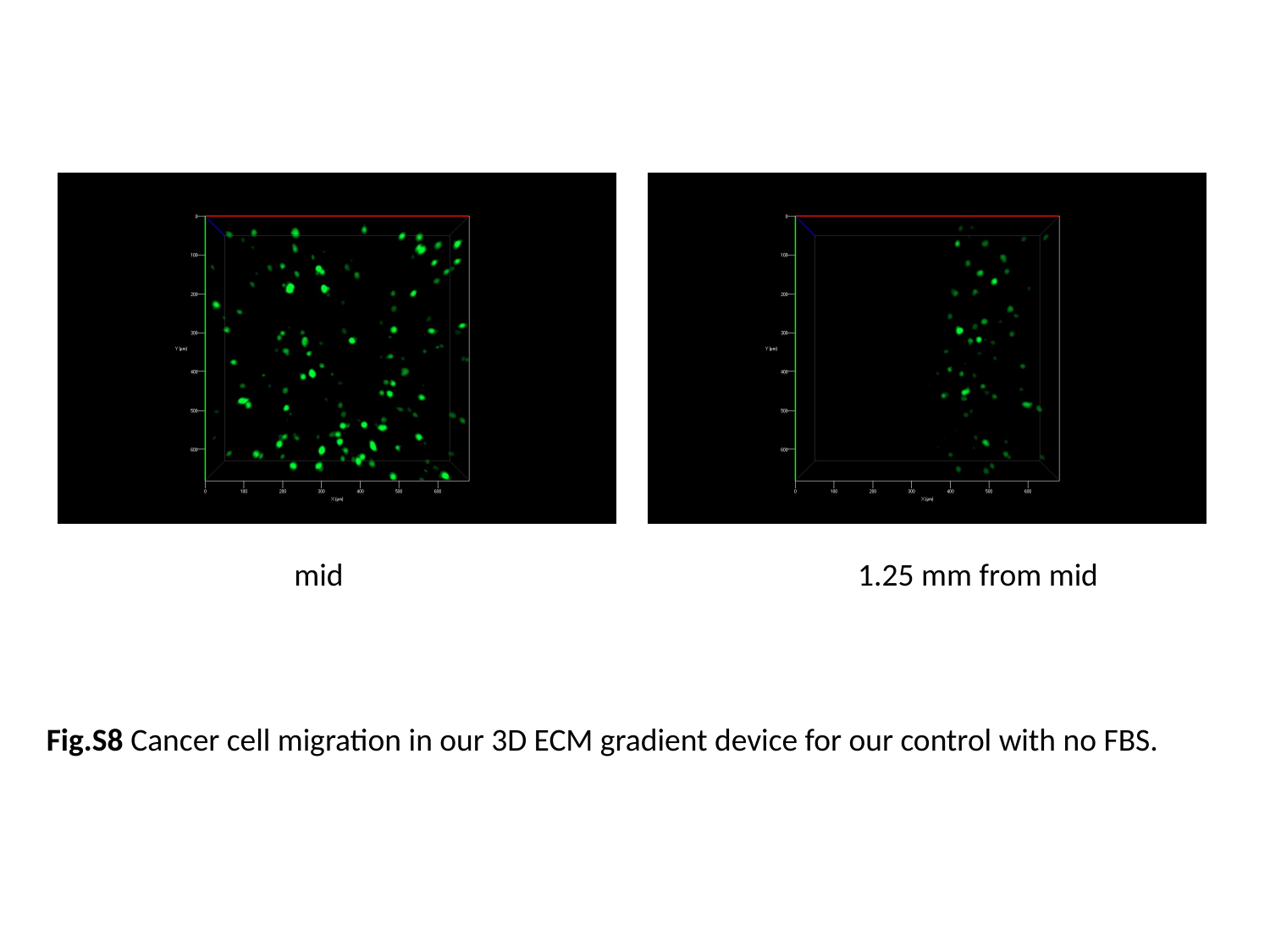

1.25 mm from mid
mid
Fig.S8 Cancer cell migration in our 3D ECM gradient device for our control with no FBS.

## Slide 10
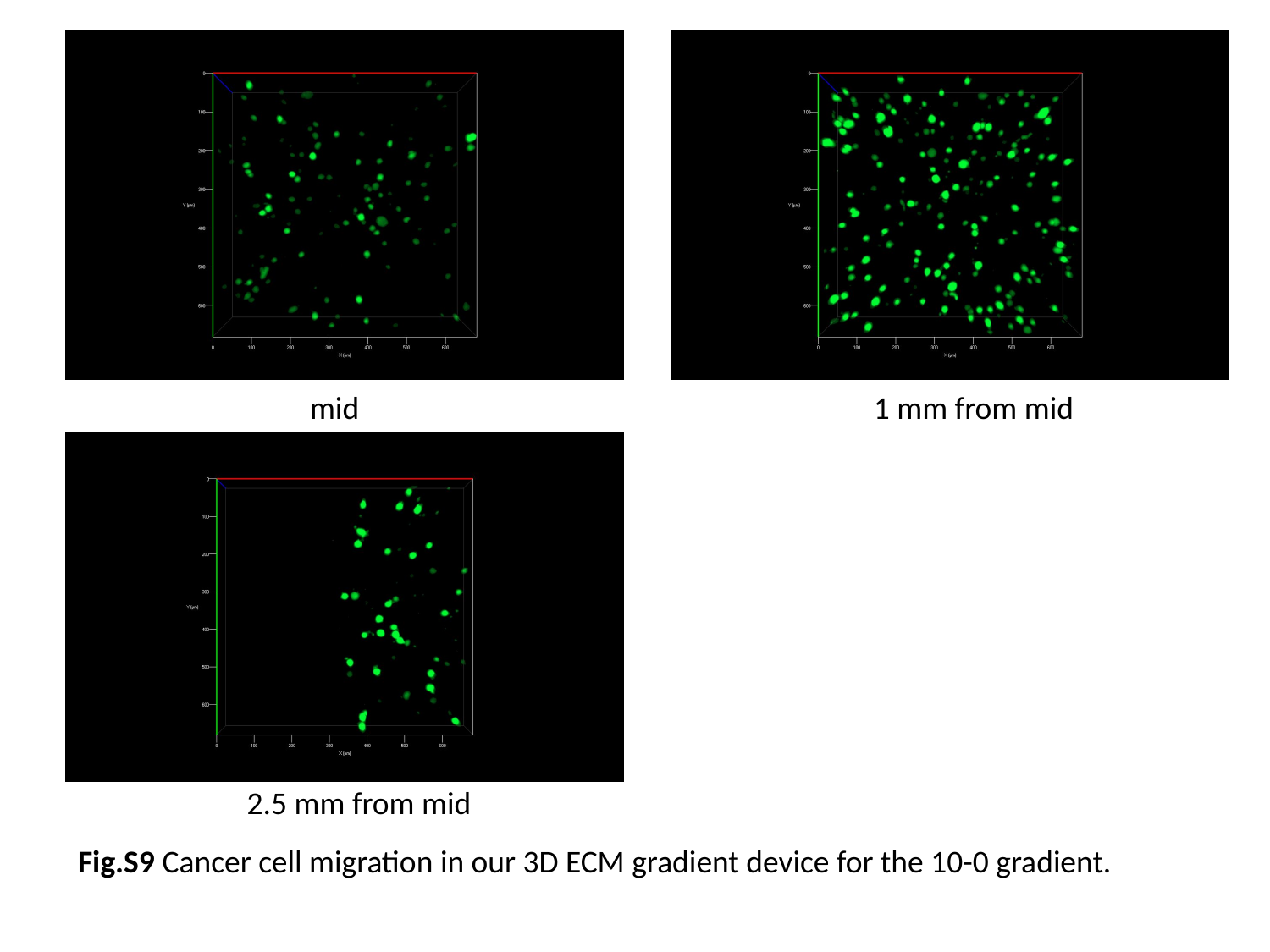

1 mm from mid
mid
2.5 mm from mid
Fig.S9 Cancer cell migration in our 3D ECM gradient device for the 10-0 gradient.

## Slide 11
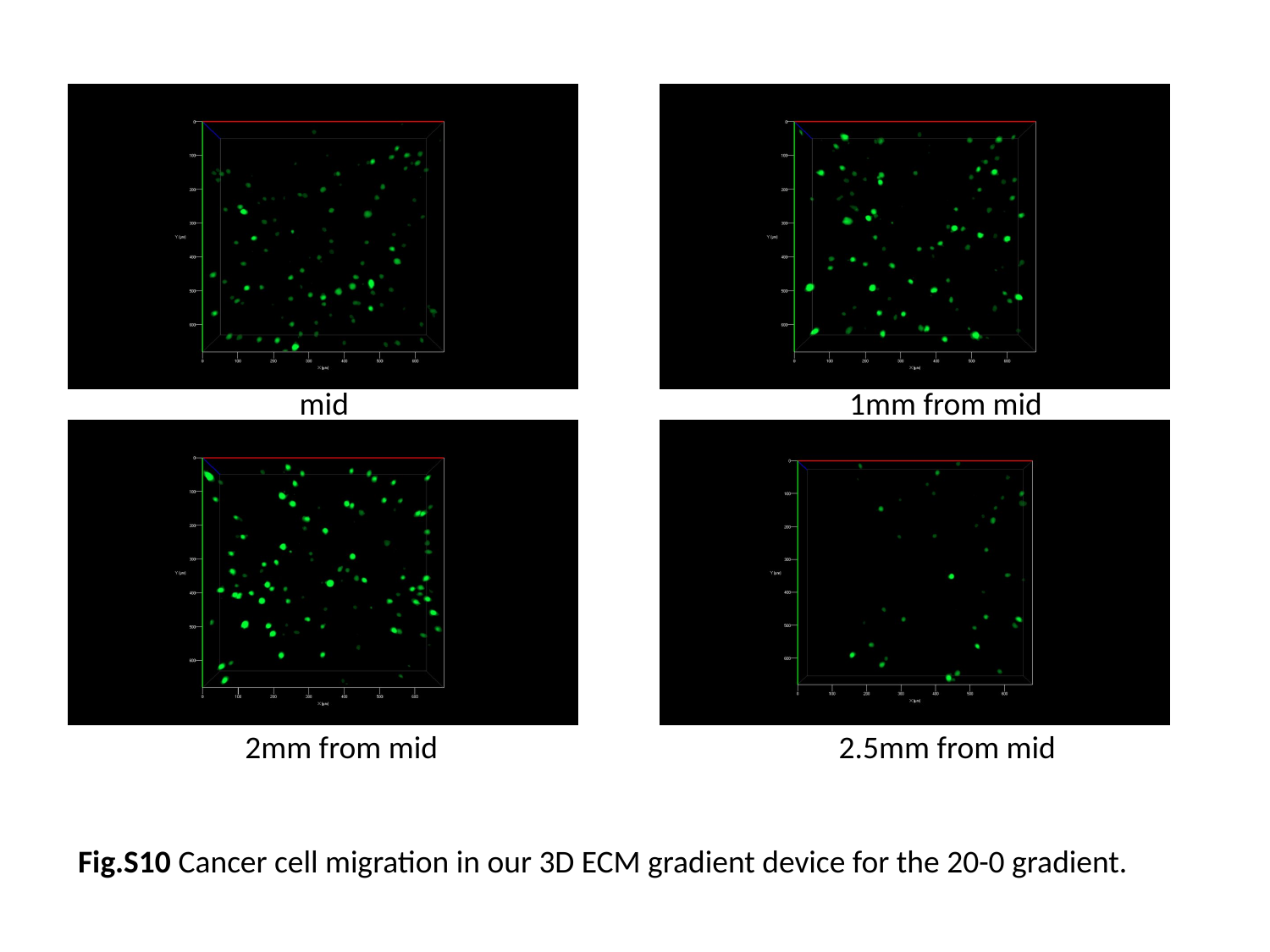

1mm from mid
mid
2mm from mid
2.5mm from mid
Fig.S10 Cancer cell migration in our 3D ECM gradient device for the 20-0 gradient.
